# Timing Matters: Post‐Stress Biostimulant Application Promotes Growth Recovery and Physiological Rebalancing in Drought‐Stressed Durum Wheat ( *Triticum durum* Desf.)

**DOI:** 10.1111/ppl.71104

**Published:** 2026-09-14

**Authors:** Matteo Spada, Moez Maghrebi, Giulia Quagliata, Eleonora Coppa, Roberto Ruggeri, Francesco Rossini, Gianpiero Vigani, Stefania Astolfi

**Affiliations:** ^1^ Department of Agriculture and Forest Sciences (DAFNE) University of Tuscia Viterbo Italy; ^2^ Department of Life Sciences and Systems Biology University of Torino Torino Italy

**Keywords:** biostimulant, Ionomic modulation, post‐stress recovery, root plasticity, stress‐responsive genes

## Abstract

As climate change intensifies drought across the Mediterranean basin, safeguarding durum wheat, a cornerstone of regional agriculture, is a global priority. This study evaluated the efficacy of a novel biostimulant, containing glycine betaine, vaterite, and *Pseudomonas protegens*, with a specific focus on a critical factor: the application timing. To distinguish preventive (pre‐stress) and restorative (post‐stress) strategies, the treatment was applied either before or after drought induction in drought‐sensitive durum wheat seedlings. Drought severely inhibited growth, increased lipid peroxidation, and altered nutrient profiles. The effect of the biostimulant was highly dependent on application timing. Pre‐stress application mainly induced tissue‐specific metabolic and oxidative responses. In contrast, post‐stress treatment promoted recovery of shoot biomass to values comparable to well‐watered controls and was associated with increased stomatal density and leaf greenness relative to untreated drought‐stressed plants. Post‐stress treatment also produced a root morphological profile closer to that of control plants and a shoot ionomic pattern more similar to well‐watered plants. Selected stress‐responsive genes showed lower transcript levels in biostimulant‐treated than in untreated drought‐stressed plants, indicating treatment‐associated changes in the expression of specific stress‐related pathways during recovery. These findings highlight post‐stress biostimulant application as a promising timing‐dependent strategy to support the recovery of drought‐sensitive durum wheat seedlings.

## 1 Introduction

Drought poses a significant threat to global food security, particularly in arid and semi‐arid regions. Its negative effects on crop growth, development, and yield are well‐documented (Dhakal 2021). Drought stress triggers morphological, physiological, and biochemical alterations that can significantly compromise plant health and productivity (Seleiman et al. 2021).

Durum wheat ( *Triticum durum* Desf.), a vital crop for pasta production worldwide (FAO 2017), is highly sensitive to drought stress, which can lead to substantial yield losses and compromised grain quality (Hinojosa et al. 2018). The Mediterranean region is the heartland of durum wheat cultivation (Martínez‐Moreno et al. 2022). This region, characterized by hot, dry summers and irregular rainfall patterns, is particularly vulnerable to climate change‐induced water scarcity. In the last decades, warming temperatures caused earlier heat and drought stress in the spring, thus reducing the tillering stage and, consequently, Mediterranean wheat's yield potential (Rossini, Ruggeri, Belocchi, and Rossini 2024).

To mitigate the negative impacts of drought on durum wheat production, innovative and sustainable agricultural practices are urgently needed. Addressing these challenges will be crucial to reach enhanced food security and the goal of sustainable crop production intensification.

In recent years, biostimulants have emerged as a promising tool to enhance plant resilience and productivity under various stress conditions, including drought. These substances of biological origin, derived from plants, seaweeds, animals, or microbial sources, stimulate plant growth and development, improve nutrient uptake, and enhance stress tolerance mechanisms (Bulgari et al. 2019; Rouphael and Colla 2020). By modulating physiological and biochemical processes, biostimulants can help plants cope with water scarcity, maintain photosynthetic efficiency, and optimize resource utilization. These positive effects occur regardless of the biostimulant's nutrient content, making biostimulants valuable tools for sustainable agricultural practices and for addressing challenges related to food production.

In our previous work, the impact of biostimulants, including plant growth‐promoting bacteria and seaweed extracts, on key physiological and biochemical processes in durum wheat under drought conditions was critically evaluated. These processes included plant growth rate, chlorophyll levels, root system architecture, stomatal regulation, and oxidative damage (Spada et al. 2024). Our results showed that while biostimulants have shown promise in mitigating drought stress in a relatively sensitive durum wheat cultivar (Iride), their efficacy on a drought‐tolerant genotype (SvEMS16) appeared to be limited. This suggests that the application of biostimulants may be most beneficial as a targeted strategy for cultivars that are inherently more susceptible to drought‐induced damage (Spada et al. 2024).

Here, we aim to shed light on the efficacy of a previously tested biostimulant composed of glycine betaine, vaterite, and *Pseudomonas protegens* in preventing or mitigating drought‐induced damage in a sensitive durum wheat cultivar. Recent germination studies showed seaweed‐based biostimulants enhanced root development from seed coating under both optimal and saline conditions (Rossini, Ruggeri, and Rossini 2024; Rossini, Ruggeri, Mzid, et al. 2024), expanding the opportunities to effectively apply biostimulants across the crop cycle, thus creating new potential strategies for durum wheat management tailored to each growing environment. Building on this, our biostimulant was sprayed on seedling leaves either before or after drought imposition, and antioxidant enzyme activities, osmolyte accumulation, ionomic profiles, and expression of drought‐responsive genes were analyzed. These analyses characterize the timing‐dependent growth, physiological, ionomic, and transcriptional responses associated with biostimulant application under drought conditions.

## 2 Materials and Methods

### 2.1 Composition and Preparation of Biostimulant

The biostimulant used in this study is an experimental product containing glycine‐betaine, vaterite, and *Pseudomonas protegens*. These active components, in mixture with seaweed extracts from *Codium fragile* , have been recently found to reduce by 33% the field application of nitrogen fertilizer (Rossini et al. 2025). Before spraying it, the biostimulant was diluted to 2% with deionized H_2_O, according to Spada et al. (2024).

### 2.2 Plant Material and Growing Condition

The seeds of durum wheat ( *Triticum durum* Desf., cv. Iride) were germinated in the dark at 28°C for 4 days. Uniform seedlings were then transferred to hydroponic culture in plastic pots (three seedlings per pot) containing 600 mL of nutrient solution (NS) (Spada et al. 2024). Plants were grown in a climate chamber under controlled conditions: light intensity of 200 μE m^−2^ s^−1^, a 16/8 h (light/dark) photoperiod, day/night air temperatures of 28°C/20°C, and 80% relative humidity.

Seven days after sowing (DAS), half of the plants were subjected to water stress by adding 10% (m/V) polyethylene glycol (PEG) 6000 to the NS, establishing control (C) and drought stress (D) conditions. A biostimulant was applied to both C and D plants via foliar spraying (320 μL per plant) either 4 days before (pre‐treatment) or 4 days after (post‐treatment) the induction of water stress, corresponding to 3 and 11 days after sowing for the control plants. At harvest (15 DAS), we obtained six different conditions: (1) C, control plants adequately supplied with water, (2) pre‐T and (3) post‐T control plants adequately supplied with water and treated with biostimulant 3 and 11 days after sowing, respectively, (4) D, plants subjected to drought, and (5) pre‐D and (6) post‐D, plants treated with biostimulant before and after drought imposition, respectively (Table 1).

**TABLE 1:** Growth conditions.

Condition	Biostimulant (μL plant^−1^)	PEG (m/V)
C	0	0
pre‐T	320	0
post‐T	320	0
D	0	10%
pre‐D	320	10%
post‐D	320	10%

*Note:* Durum wheat seedlings were grown for 15 days under different growth conditions.

Abbreviations: C, control plants (adequately supplied with water); D, plants subjected to drought; pre‐D and post‐D, plants treated with biostimulant before and after drought imposition, respectively; T, control plants treated with biostimulant.

For each treatment, three independent hydroponic pots were used as biological replicates. Each pot contained three seedlings grown in the same nutrient solution and therefore represented one independent growth unit. Seedlings within the same pot were not treated as independent biological replicates.

### 2.3 Determination of Leaf Greenness

Leaf greenness was non‐destructively estimated at harvest (15 DAS) using a portable chlorophyll meter (SPAD‐502 Plus, Konica Minolta). Measurements were taken on the youngest fully expanded leaf and expressed in SPAD units.

### 2.4 Determination of Stomatal Density

The stomatal density was determined 15 DAS using the first leaf sampled from each independent biological replicate (*n* = 3). Because each biological replicate corresponded to one hydroponic pot, microscope fields obtained from the same replicate were treated as technical subsamples. Following the silicone rubber impression method, transparent synthetic enamel was applied to the abaxial (lower) leaf surface. Once dry, the impressions were collected using transparent adhesive tape and mounted onto microscope slides. Images were captured using an Axioskop 2 Plus optical microscope equipped with dedicated acquisition and processing software. For each replicate, three representative fields were photographed at 400× magnification, totaling nine images per treatment. Stomatal density was determined by manual counting and expressed as the number of stomata per square millimeter (stomata/mm^2^).

### 2.5 Analysis of Root Morphology

Root morphological traits were assessed at harvest (15 DAS). Roots were separated from shoots, placed in a plexiglass tray with a thin water layer to prevent overlap, and imaged. Root architecture was analyzed with WinRHIZO software (Regent Instruments Inc.) to quantify total length, surface area, volume, average diameter, and tip number (Spada et al. 2024).

### 2.6 Measurement of Malondialdehyde Concentration

Malondialdehyde (MDA), a primary end product of lipid peroxidation, was determined using the thiobarbituric acid (TBA) assay to estimate the extent of oxidative stress, according to Quagliata et al. (2023). Fresh shoot and root tissues (0.2 g) were homogenized in 2 mL of 0.25% TBA in 10% TCA. The extract was heated at 95°C for 30 min and then rapidly cooled on ice. After centrifuging the sample at 16,000×*g* for 20 min, the absorbance of the supernatant was measured at 532 nm. To correct for nonspecific turbidity, the absorbance recorded at 600 nm was subtracted. The level of lipid peroxidation was then expressed as μg g^−1^ fresh weight (FW), using an extinction coefficient of 155 mM cm^−1^.

### 2.7 Measurement of Proline Concentration

The proline concentration was estimated as described by Quagliata et al. (2023) following the classical acid‐ninhydrin colorimetric assay originally described by Bates et al. (1973). Root and shoot tissues (0.1 g FW) were homogenized in 2 mL of 3% (w/v) sulfosalicylic acid and centrifuged at 3000×*g* for 10 min. The resulting supernatant was used for proline quantification. An aliquot of the extract was mixed with 500 μL of acid‐ninhydrin reagent and 500 μL of glacial acetic acid, and the reaction mixture was incubated at 100°C for 1 h. The reaction was then terminated by cooling on ice, and the chromophore was extracted with 1.5 mL of toluene. Absorbance was measured at 520 nm, and proline concentration was expressed as μmol g^−1^ fresh weight (FW).

### 2.8 Antioxidant Enzyme Extraction and Activity

Catalase (CAT; E.C. 1.11.1.6) activity was assessed by evaluating the disappearance of H_2_O_2_ at 240 nm (Agilent Cary 3500 UV–Vis spectrophotometer), as described by Quagliata et al. (2024). Peroxidase (POD; E.C. 1.11.1.7) activity was measured spectrophotometrically (Agilent Cary 3500 UV–Vis spectrophotometer) at 470 nm using guaiacol as a hydrogen donor, according to Coppa et al. (2024). The resulted extracts were used for the evaluation of soluble protein content in accordance with the method of Bradford (1976), using bovine serum albumin (BSA) as standard.

Both enzyme activities were linear with time and proportional to the amount of extract used.

### 2.9 Ionomic Composition Analysis of Plant Tissues

Shoot and root tissues were oven‐dried at 80°C for 24 h and then digested using a microwave digestion system (Multiwave Go Plus, Anton Paar GmbH). For digestion, 0.120 g (shoots) and 0.07 g (roots) of dry weight (DW) were processed with 3 mL of 69.5% HNO_3_ and 0.6 mL of 37% HCl. The resulting digests were filtered, diluted 200‐fold with Milli‐Q H_2_O, and subsequently analysed by ICP‐MS (Agilent 7850 ICP‐MS) to determine the concentrations of magnesium (Mg), potassium (K), calcium (Ca), copper (Cu), manganese (Mn), iron (Fe), and zinc (Zn).

### 2.10 Gene Expression Analysis

Total RNA was extracted from roots and shoots using TRIzol Reagent (Life Technologies) and then purified using PureLink RNA Mini Kit (Life Technologies), according to the manufacturer's instructions. Contaminant DNA was removed on‐column using PureLink DNase (Life Technologies). First‐strand cDNA synthesis was carried out using the SuperScriptTM III First‐Strand Synthesis SuperMix for qRT‐PCR (Invitrogen), according to the manufacturer's instructions.

qRT‐PCR analysis of drought‐responsive genes (*TdP5CS*, *TdDHN15.3*, *TdDRF1*, *TdSHN1*, and *TdWRKY2*) was performed on first‐strand cDNA in a 20 μL reaction mixture containing GoTaq qPCR Master Mix (Promega) and the specific primers (Quagliata et al. 2024), using the QuantStudio 3 Real‐Time PCR System (Applied Biosystems). The relative transcript level of each gene was calculated by the 2^−ΔΔCt^ method (Livak and Schmittgen 2001) using the expression of the *TdACTIN* gene as a reference.

### 2.11 Statistical Analysis

Each reported value represents the mean ± standard deviation (SD) of measurements performed in triplicate and obtained from three independent biological replicates (*n* = 3), each corresponding to one hydroponic pot. Data were analyzed using two‐way ANOVA to evaluate the effects of drought stress, biostimulant application timing, and their interaction on the measured variables. The analysis was carried out using R software (Rstudio, V. 2023.12.0 + 369) and the packages: ggplot2 (version 3.5.1), dplyr (version 1.1.4), tidyverse (version 2.0.0), car (version 3.1‐3), and agricolae (version 1.3‐7). To identify significant differences among growth conditions, one‐way ANOVA followed by Tukey's post hoc test was applied at *p* < 0.05. Hierarchical clustering on shoot and root traits and ionomic datasets was performed with the full method and Euclidean distance measurement in R (V. 2023.12.0 + 369), using the packages pheatmap (version 1.0.12), RColorBrewer (version 1.1–3), and gplots (version 3.1.3.1).

## 3 Results

As the timing of the biostimulant treatment in control plants had a negligible effect on most traits, except for gene expression, the pre‐T and post‐T conditions were combined and represented as a single histogram (T) in most graphs.

### 3.1 Effect of Biostimulant Treatments and Drought Stress on Plant Growth

**FIGURE 1:**
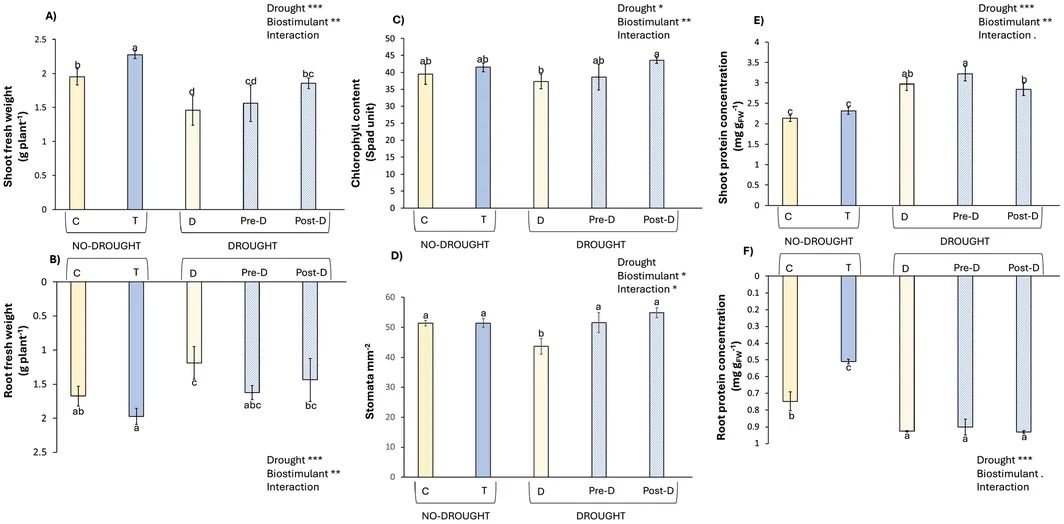
Impact of drought stress and biostimulant application timing on shoot (A) and root (B) fresh biomass, leaf chlorophyll content (C), stomatal density (D), and shoot (E) and root (F) protein concentration in durum wheat seedlings grown for 15 days under different growth conditions: C, control plants (adequately supplied with water); D, plants subjected to drought; pre‐D and post‐D, plants treated with biostimulant before and after drought imposition; T, control plants treated with biostimulant, respectively. Each reported value represents the mean ± standard deviation (SD) of measurements performed in triplicate and obtained from three independent biological replicates (*n* = 3). Means sharing the same letter are not significantly different according to Tukey's HSD test (*p* < 0.05). Data were also analyzed using two‐way ANOVA (table in each plot).

In well‐watered wheat plants (C), biostimulant application significantly increased shoot biomass by 17% compared to the untreated control (Figure 1A).

Water stress significantly inhibited shoot and root growth by 25% and 29%, respectively. However, post‐stress biostimulant treatment partially mitigated the negative effects of drought on shoot biomass, restoring it to levels comparable to the control (Figure 1A,B). Notably, chlorophyll content remained unchanged across all drought conditions and biostimulant applications; however, a significant 17% increase was observed in the post‐D condition compared to the D one (Figure 1C).

Drought stress significantly reduced stomatal density by 15% relative to well‐watered controls (Figure 1D). Conversely, biostimulant application, whether applied before (pre‐D) or after (post‐D) the onset of stress, mitigated this effect. Specifically, stomatal density increased by 18% and 26%, respectively, compared to untreated drought‐stressed plants (Figure 1D).

Biostimulant application to control plants reduced root protein concentration by 32% (Figure 1F). Conversely, water stress increased shoot and root protein concentrations by 39% and 24%, respectively, compared to the control (Figure 1E,F). Neither pre‐ nor post‐stress biostimulant treatments significantly affected protein concentration in drought‐stressed plants (Figure 1E,F).

**FIGURE 2:**
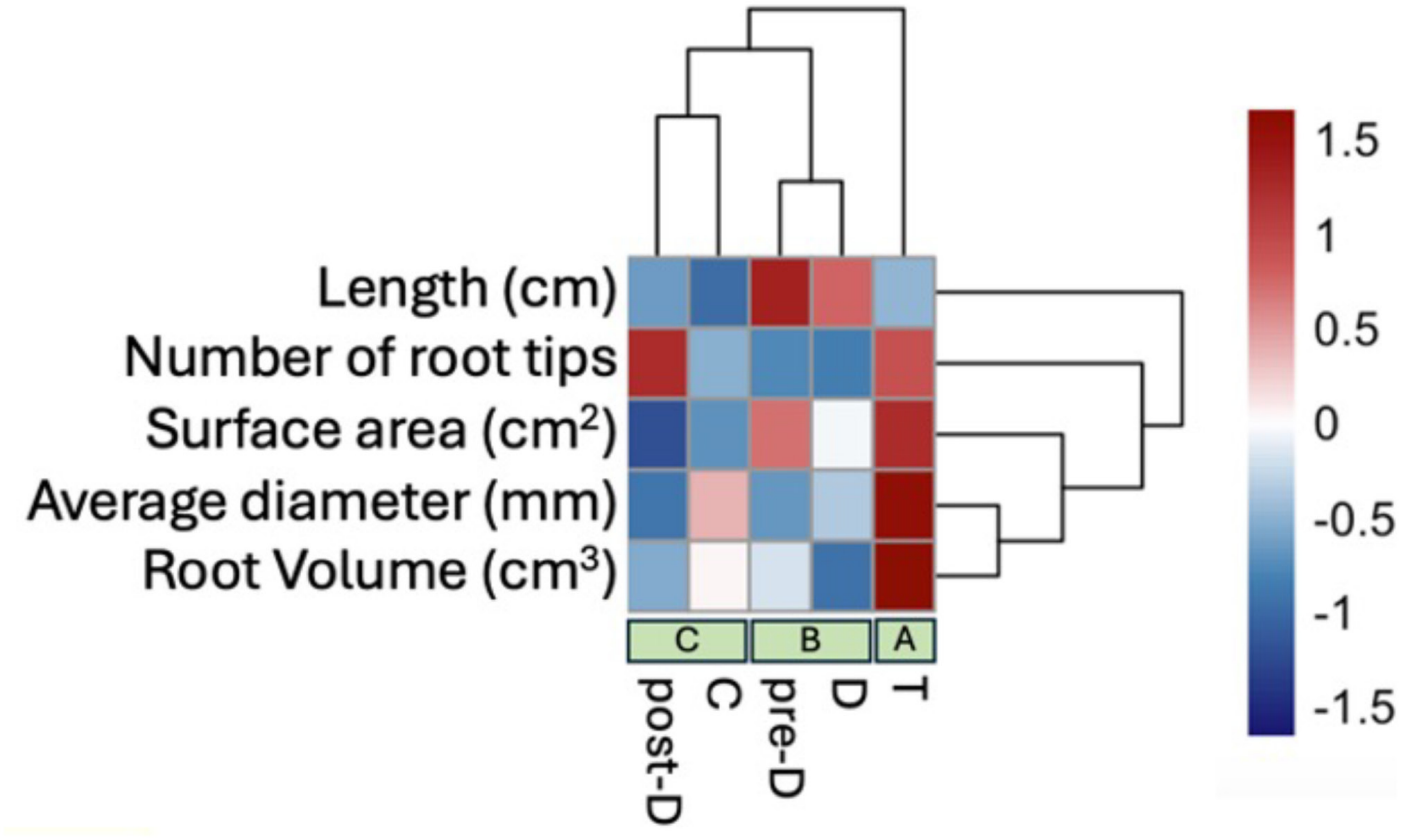
Heatmap showing the hierarchical clustering of data related to morphological parameters of the root system of durum wheat seedlings, obtained using the complete method and Euclidean distance. The color bar represents the gradient values for root traits.

To further investigate treatment effects on plant growth, root morphology (length, volume, diameter, area, and number of tips) was analyzed under different growing conditions (Figure S1). Hierarchical clustering and heatmap analysis of the complete root traits dataset grouped the five different growth conditions into three distinct clusters (Figure 2). Cluster B grouped the pre‐D and D conditions, indicating similar root architecture. Cluster C comprised the C and post‐D conditions, while the T condition formed a separate cluster (A), suggesting a distinct morphological profile (Figure 2). Specifically, well‐watered plants treated with biostimulant (T) exhibited thicker roots and a higher root number compared to the control (C). Under drought stress (D), plants developed longer roots with a larger surface area than control plants (C) (Figure 2). While pre‐stress biostimulant application (pre‐D) did not substantially modify the root morphological profile compared with drought‐stressed plants (D), post‐stress treatment (post‐D) shifted the overall root trait profile toward that of well‐watered control plants (Figure 2). This response was particularly evident in the clustering analysis and in the higher number of root tips.

### 3.2 Physiological and Molecular Response to Biostimulant Treatments and Drought Stress

**FIGURE 3:**
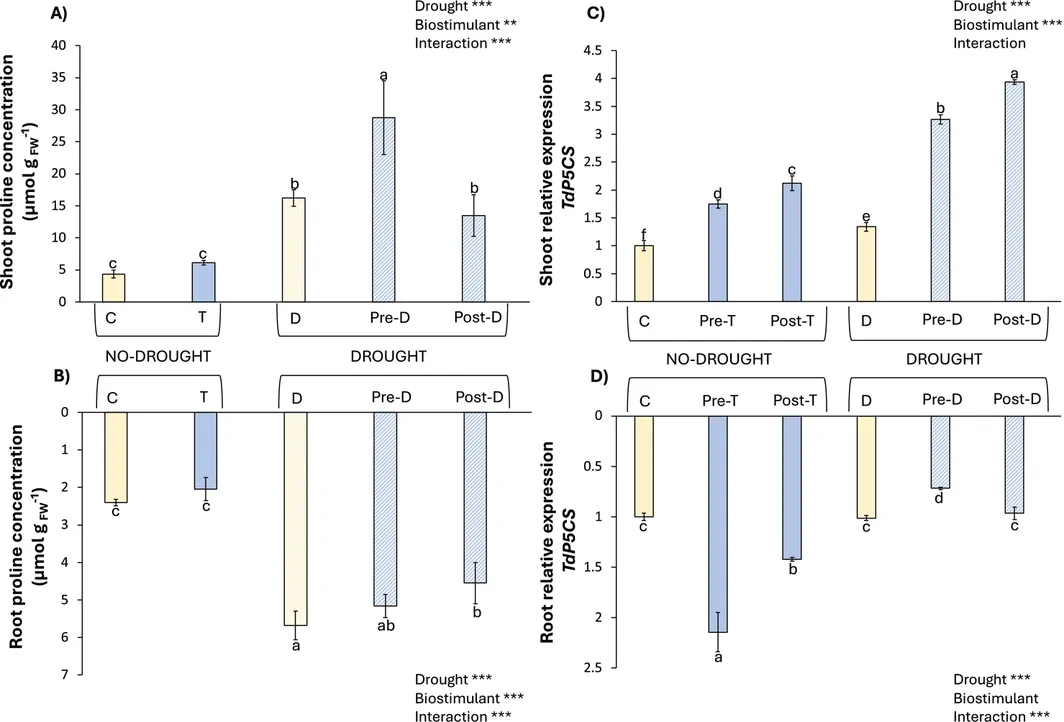
Shoot (A) and root (B) proline concentration and shoot (C) and root (D) relative expression of TdP5CS gene in durum wheat seedlings exposed to the various growth conditions described in Figure 1. Statistics as in Figure 1.

The molecular response to biostimulant treatments under drought stress involved complex tissue‐specific and timing‐dependent regulatory mechanisms. Drought stress triggered substantial proline accumulation (274 and 136% in shoots and roots, respectively) through activation of *TdP5CS* gene encoding pyrroline‐5‐carboxylate synthase, a pivotal enzyme in the synthesis of proline (Turchetto‐Zolet et al. 2008). Biostimulant application to control plants did not significantly affect proline content (Figure 3A,B). Interestingly, the timing of biostimulant application to drought‐stressed plants differentially affected proline accumulation. Pre‐stress biostimulant application resulted in a 77% increase in shoot proline, while post‐stress treatment led to a 20% decrease in root proline, both compared to plants experiencing drought stress alone (D) (Figure 3A,B).

The study reveals complex transcriptional reprogramming. Shoot expression of the *TdP5CS* gene increased with both drought and biostimulant applications, indicating synergistic activation of proline biosynthesis pathways (Figure 3C). In roots, expression only raised in well‐watered control plants receiving biostimulant, with drought conditions suppressing this response (Figure 3D).

Beyond proline biosynthesis, a complex network of genes plays a crucial role in maintaining drought tolerance. Key players in this response include dehydrins, such as those encoded by *TdDHN15.3*, which are involved in protecting cellular structures under stress conditions. Additionally, various transcription factors (TFs) such as *TdDRF1*, *TdSHN1*, and *TdWRKY2* orchestrate the expression of various drought‐responsive genes (Rampino et al. 2006; He et al. 2016; Zhang et al. 2022; Maghrebi et al. 2024).

**FIGURE 4:**
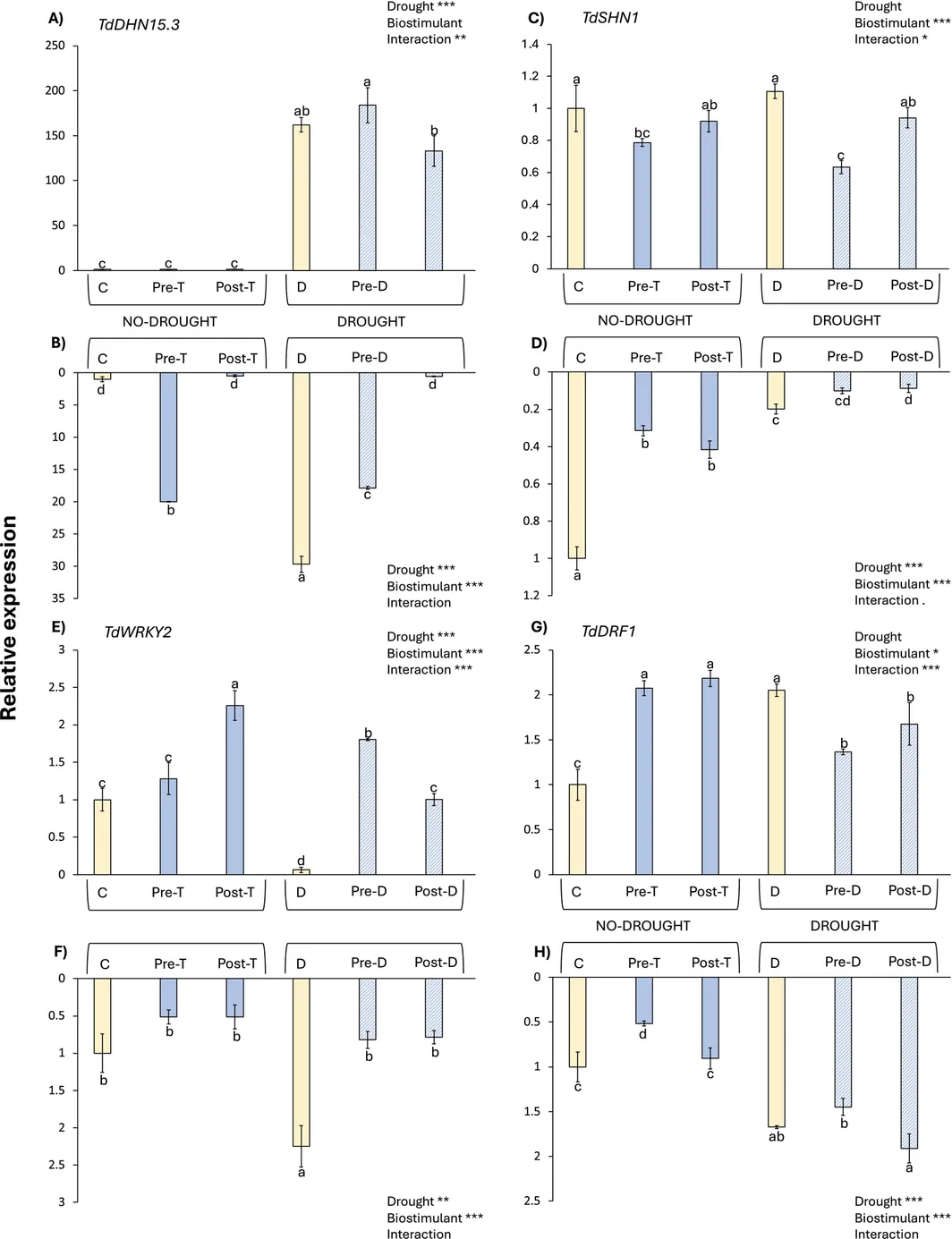
Shoot and root relative expression of TdDHN15.3 (A, B), TdSHN1 (C, D), TdWRKY2 (E, F) and TdDRF1 (G, H) genes in durum wheat seedlings exposed to the various growth conditions described in Figure 1. Statistics as in Figure 1.

Regarding the *TdDHN15.3* gene, relative transcript levels significantly increased under drought stress at both shoot and root levels (Figure 4A,B). At the root level, its expression was also increased in the pre‐treatment control condition (pre‐T). In contrast, when the biostimulant was applied to drought‐stressed plants, either before (pre‐D) or after (post‐D) stress imposition, *TdDHN15.3* expression in roots decreased compared to untreated drought‐stressed plants (D) (Figure 4B). Drought stress differentially affected the expression of the *TdWRKY2* transcription factor, causing a decrease in shoots but an increase in roots (Figure 4E,F). Biostimulant application further modulated *TdWRKY2* expression: it increased in shoots in the post‐treatment control (post‐T), and in both pre‐D and post‐D conditions (Figure 4E). Conversely, at the root level, *TdWRKY2* expression decreased when the biostimulant was applied to stressed plants (pre‐D, post‐D) (Figure 4F). Water stress consistently increased the relative transcript levels of the *TdDRF1* gene in both plant tissues (Figure 4G,H). At the shoot level, biostimulant application in well‐watered control plants (pre‐T and post‐T) further increased *TdDRF1* expression (Figure 4G). Conversely, in drought‐stressed plants, biostimulant application (pre‐D and post‐D) led to a decrease in its expression in shoots (Figure 4G). At the root level, *TdDRF1* expression was reduced in the pre‐treatment control (pre‐T) condition (Figure 4H). Regarding the *TdSHN1* gene, transcript levels in shoots were reduced in both the pre‐treatment control (pre‐T) and pre‐stress drought (pre‐D) conditions (Figure 4C). In roots, *TdSHN1* expression was decreased by water stress (Figure 4D). Additionally, biostimulant application to control plants (pre‐T and post‐T) and post‐stress application to drought‐stressed plants (post‐D) also resulted in reduced *TdSHN1* expression in roots (Figure 4D).

### 3.3 Oxidative Damage and Antioxidant Activities in Response to Biostimulant Treatments and Drought Stress

Drought stress commonly triggers the overproduction of reactive oxygen species (ROS), leading to lipid peroxidation, which is frequently assessed by measuring malondialdehyde (MDA) levels (Tirani and Haghjou 2019).

**FIGURE 5:**
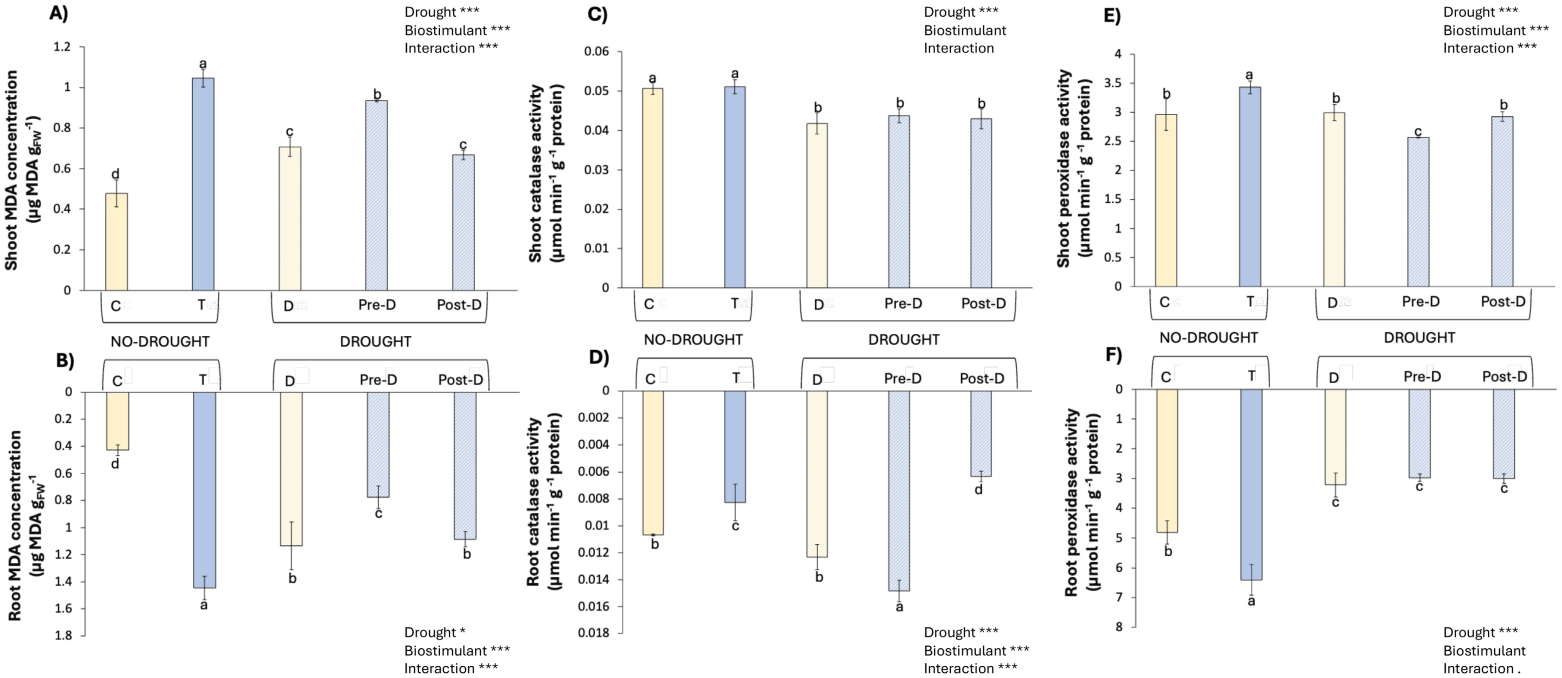
Shoot and root MDA (A, B) concentrations, catalase (C, D) and peroxidase (E, F) activity in wheat seedlings exposed to the various growth conditions described in Figure 1. Statistics as in Figure 1.

Biostimulant application to control plants significantly increased MDA levels in both shoots and roots, by 119% and 238%, respectively (Figure 5A,B). Similarly, water stress induced a significant increase in MDA concentration in both tissues, with a 48% increase in shoots and a 148% increase in roots (Figure 5A,B). Regarding the different biostimulant application timings under drought, only the pre‐stress application significantly modulated MDA levels. In drought‐stressed plants, this pre‐treatment led to a 32% increase in shoot MDA but, conversely, a 32% decrease in root MDA level when compared to drought‐stressed plants without biostimulant treatment (D) (Figure 5A,B).

To counteract oxidative stress from environmental factors like water deficit, plants activate antioxidant enzymes such as catalase (CAT) and peroxidase (POD) (Cao et al. 2017).

In well‐watered control plants, biostimulant application significantly reduced root CAT activity by 23% (Figure 5D). Conversely, it significantly increased both shoot and root POD activity by 16% and 33%, respectively (Figure 5E,F). The imposition of water stress significantly decreased shoot CAT activity by 17% and root POD activity by 33% (Figure 5C,F). Biostimulant applications had a significant and differential impact on antioxidant enzyme activity in drought‐stressed plants. Pre‐stress treatment significantly increased root CAT activity by 20% and significantly decreased shoot POD activity by 14% compared to untreated drought‐stressed plants (D) (Figure 5E). In contrast, post‐stress treatment significantly decreased root CAT activity by 49% in comparison to drought‐stressed plants (D) (Figure 5D).

### 3.4 Ionomic Modulation of Shoot and Root Tissues

**FIGURE 6:**
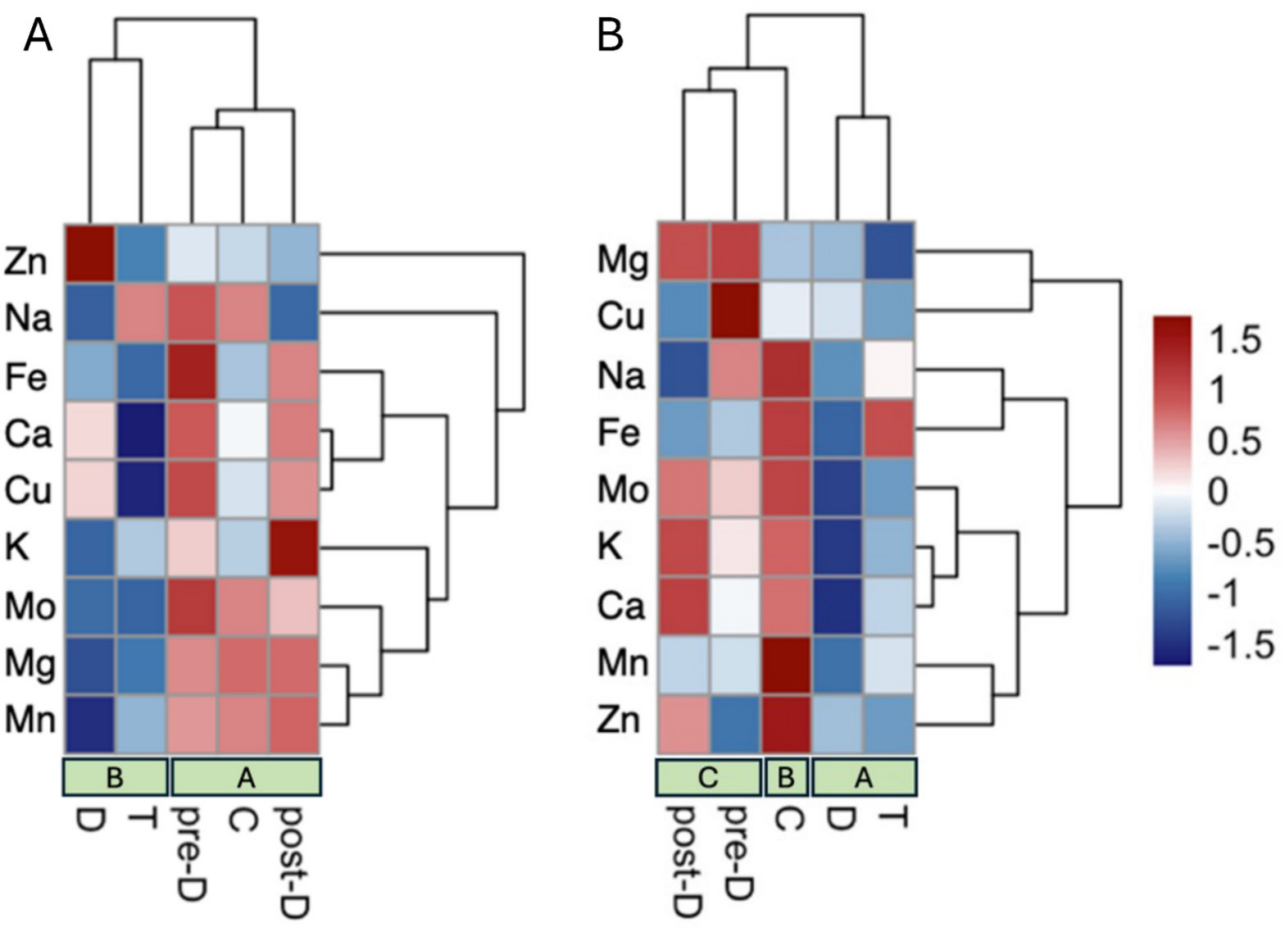
Heatmap showing the hierarchical clustering of data related to ionomic concentration of shoot (A) and root tissues (B) of durum wheat seedlings, obtained using the complete method and Euclidean distance. Growth conditions as in Figure 1. The color bar represents the gradient values for shoot and root traits.

To comprehensively evaluate the effects of biostimulant treatments and water stress on plant mineral nutrient profiles, we conducted an ionomic analysis of shoot and root tissues (Figure S2). Hierarchical clustering of the ionomic data provided valuable insights into how these treatments influenced the accumulation and distribution of mineral nutrients within the plant (Figure 6).

In shoots, the drought stress (D) and biostimulant‐treated control (T) groups clustered together (Cluster B), resulting in a similar pattern of reduced accumulation for most of the analysed nutrients (Figure 6A). Notably, Zn showed the highest relative concentration in the drought‐stressed plants (D), suggesting a potential accumulation under water deficit. On the other hand, the pre‐ and post‐stress biostimulant application under drought (pre‐D and post‐D) and the control (C) groups clustered together (Cluster A), revealing that applying the biostimulant to drought‐stressed plants, regardless of whether it is before or after the stress, led to a shoot ionome profile that is more similar to the well‐watered control plants than to the plants experiencing drought alone (Figure 6A). This suggests that biostimulant application partially mitigated drought‐induced changes in the shoot ionomic profile. However, the root ionomic response followed a different clustering pattern, indicating tissue‐specific nutrient modulation rather than a uniform restoration of whole‐plant ionomic homeostasis.

Indeed, the root ionomic profile revealed the drought stress (D) and biostimulant‐treated control (T) groups clustered together (Cluster A) (Figure 6B). However, in this tissue, the control (C) condition formed its own distinct cluster (Cluster B), while the pre‐ and post‐stress biostimulant application under drought (pre‐D and post‐D) groups clustered together (Cluster C) (Figure 6B).

## 4 Discussion

The Mediterranean basin, responsible for approximately 60% of global durum wheat (DW) production (FAO 2017), is facing a severe water crisis, affecting over 180 million people (COP29). The strong dependence of durum wheat on rainfall makes it highly vulnerable to drought, leading to significant yield fluctuations (Hinojosa et al. 2018).

### 4.1 Physiological and Molecular Plant Adaptation to Drought Stress

Drought stress reduced plant growth, decreasing shoot and root biomass by 25% and 29%, respectively (Figure 1A,B). This inhibition is commonly associated with reduced turgor, stomatal closure limiting CO_2_ uptake, and impaired photosynthesis (Fahad et al. 2017; Haghpanah et al. 2024). Accordingly, stomatal density declined by 15% under water deficit (Figure 1D). In contrast, roots displayed marked morphological plasticity, with increased length and surface area (Figure 2), likely enhancing water and nutrient foraging under limited moisture (Comas et al. 2013). Proline levels also rose markedly in shoots and roots (Figure 3A,B), contributing to osmotic adjustment and protection against dehydration (Verbruggen and Hermans 2008; Szabados and Savouré 2010), consistent with the induced expression of *TdP5CS*, coding a key enzyme in proline biosynthesis (Amini et al. 2015; Funck et al. 2020) (Figure 4C). Drought further triggered transcriptional reprogramming in roots, with upregulation of *TdDRF1* (DREB family) and *TdWRKY2* (Figure 4F,H), both central regulators of downstream drought‐responsive genes involved in osmolyte accumulation and stress adaptation (Liu et al. 2022; Rushton et al. 2010; Niu et al. 2012; Maghrebi et al. 2024). Accordingly, their upregulation in roots highlighted a systemic regulatory effort to reprogram gene expression, leading to the observed morphological and physiological adaptations to cope with water scarcity.

Drought induced severe oxidative stress, with MDA increasing by 48% in shoots and 148% in roots (Figure 5A,B), alongside reduced CAT and POD activities (Figure 5C–F), revealing limited antioxidant capacity in the Iride cultivar (Spada et al. 2024). Ionomic analysis showed a generalized decline in mineral accumulation in both organs (Figure 6A,B), likely due to impaired uptake under reduced transpiration (Quagliata et al. 2025).

Overall, drought imposed a coordinated physiological and molecular response characterized by growth suppression, osmotic adjustment, transcriptional reprogramming, and oxidative imbalance. Despite the activation of protective mechanisms, the combined oxidative damage and reduced nutrient acquisition highlight the substantial vulnerability of the Iride cultivar to prolonged water deficit.

### 4.2 Biostimulant‐Induced Growth Enhancement and Altered Nutrient and Oxidative Status in Well‐Watered Wheat

Biostimulants are widely recognized for their ability to enhance plant growth and resilience in both normal and stressful environments (Du Jardin 2015; Santaniello et al. 2017; Shukla et al. 2019). Consistent with the reported growth‐promoting effects of several biostimulants, application of the tested formulation increased shoot biomass by 17% in well‐watered wheat plants compared with untreated control (Figure 1A), likely suggesting an enhanced photosynthetic rate despite unchanged chlorophyll levels (Figure 1C); photosynthetic and water–relation parameters were not measured. Biostimulant‐treated plants (T) also developed thicker roots with a greater number of root tips (Figure 2), indicating that the treatment modified root morphology under well‐watered conditions, thereby improving potential for below‐ground resource acquisition, consistent with earlier observations for *Pseudomonas*‐based biostimulants (Zhang et al. 2023).

Despite the increase in shoot biomass, treated plants showed lower concentrations of several mineral nutrients (Figure 6). This pattern may partly reflect a growth‐dilution effect, in which biomass accumulation increases more rapidly than nutrient accumulation, although changes in nutrient uptake, transport, or tissue partitioning may also have contributed. Since biostimulants are often more effective under suboptimal conditions, including water or nutrient limitation (Li et al. 2022), the reduction observed under well‐watered conditions suggests a complex response that warrants further investigation to determine whether it is transient or reflects a more persistent change in nutrient allocation.

Although the biostimulant promoted shoot growth, its application to well‐watered plants induced a complex root response, characterized by reduced protein concentration (Figure 1F), elevated MDA levels (Figure 5B), decreased catalase (CAT) activity (Figure 5D), and increased peroxidase (POD) activity (Figure 5F). Together, these changes indicate that the treatment altered root oxidative status even in the absence of drought. A similar transient modulation of oxidative metabolism under optimal conditions was reported by Cerruti et al. (2024), who observed an early increase in H_2_O_2_ accompanied by reduced antioxidant enzyme activities, followed by H_2_O_2_ normalization and increased CAT activity. Previous studies have also suggested that colonization by *Pseudomonas* may induce an initial oxidative signal associated with ISR priming (Verhagen et al. 2009), while increased metabolic activity linked to growth promotion may also contribute to transient ROS production. However, these mechanisms were not directly assessed in the present study. The increase in POD activity may therefore represent a compensatory response to the altered oxidative status, although this interpretation remains tentative. Consistently, the modulation of stress‐responsive genes in well‐watered plants further indicates that biostimulant application triggered broader physiological adjustments beyond its effects under drought.

### 4.3 Timing‐Dependent Biostimulant Effects: Preventive Treatment Versus Post‐Stress Recovery

Biostimulants are increasingly recognized as tools for improving plant performance under abiotic stress, but their effects may depend strongly on the timing of application. In the present study, the experimental design allowed us to distinguish two biologically different scenarios: a preventive treatment applied before drought imposition and a restorative treatment applied after drought induction. The results indicate that these two timings should not be interpreted as equivalent. Overall, the evidence more strongly supports a recovery‐promoting role of post‐stress application than a preventive effect of pre‐stress application.

Under drought, post‐stress biostimulant application promoted recovery of shoot biomass to values statistically comparable with well‐watered controls (Figure 1A). This response was accompanied by higher leaf greenness (Figure 1C) and increased stomatal density (Figure 1D) compared with untreated drought‐stressed plants, indicating enhanced water‐use efficiency and active physiological adjustment. Consequently, the treatment appears to facilitate both biomass preservation and structural modifications that support sustained gas exchange as the plant recovers from stress. *P. protegens*, a key component of the biostimulant product, probably drove these growth benefits, as a plant growth‐promoting bacterium (PGPB) that produces phytohormones (especially auxins) and modulates hormonal balance to stimulate root and shoot growth under stress (Bakaeva et al. 2022). However, because stomatal density increased under both pre‐ and post‐stress applications, this trait should be interpreted as a general biostimulant‐responsive anatomical adjustment rather than a response specifically explaining the post‐stress recovery. Similarly, root morphology showed a clear timing‐dependent pattern: pre‐D clustered close to untreated drought‐stressed plants, whereas post‐D clustered with the well‐watered control condition (Figure 2). This suggests that post‐stress application favored root architectural reconfiguration during recovery, while pre‐stress application did not prevent the establishment of a drought‐like root phenotype, characterized by typical suppression of total root growth despite adaptive root/shoot ratio increases (Comas et al. 2013). This aligns with Skalski et al. (2024), who showed polyglutamic acid (γ‐PGA) enhanced aboveground biomass and root elongation in drought‐stressed grasslands by improving water and nutrient acquisition capacity (Gowtham et al. 2022).

The observed morphological benefits, such as increased root tips and improved shoot recovery, could likely be attributed to the biostimulant's modulation of growth‐promoting hormones, which improve resource acquisition and stress adaptation. Biopolymer‐based biostimulants profoundly alter root metabolic profile and trigger brassinosteroid biosynthesis (Lucini et al. 2018), while interactions with abscisic acid (ABA), cytokinins, and gibberellins, orchestrate root development. PGPR‐based biostimulants further enhance root growth by modulating indole‐3‐acetic acid (IAA) catabolism into indole‐3‐carboxylic acid (ICA) and related intermediates (Lephatsi et al. 2022). This modulation could be beneficial under stress as reduced IAA levels stimulate root elongation, improving water and nutrient uptake (Sanchez‐Calderon et al. 2013). In wheat, inoculation with *P. protegens* has been shown to increase the IAA/ABA ratio and prevent stress‐induced root growth inhibition, likely explaining the thicker, more branched roots as well as the recovery of shoot biomass observed in our treated plants after drought. In addition, glycine betaine complemented these effects by protecting photosynthesis, acting as a chloroplast osmoprotectant that stabilizes Rubisco and photosystem II while maintaining turgor for sustained shoot growth under stress (Basit et al. 2025). Finally, vaterite (CaCO_3_) supplied essential calcium to strengthen cell walls, stabilize membranes and support cell expansion under water stress (Akhtar et al. 2022). This synergy, *P. protegens* (hormonal growth promotion), glycine betaine (photosynthetic protection), and Ca (structural support), preserved shoot biomass and root architecture despite drought; this is consistent with recent findings showing that combining microbial and osmoprotectant agents can help plants maintain growth under abiotic stress by orchestrating improvements in water uptake, cell integrity, and metabolic activity (Bakaeva et al. 2022).

The oxidative stress markers were also timing‐ and tissue‐dependent. Drought sharply increased MDA (48% shoots, 148% roots) (Figure 5B) and depressed antioxidant enzyme activities (CAT, POD) in untreated plants (Figure 5). Under drought, only pre‐stress application significantly affected MDA levels, increasing MDA in shoots but decreasing it in roots, where we also found increased CAT activity. In contrast, post‐stress treatment did not significantly reduce MDA and even decreased root CAT activity compared with untreated drought‐stressed plants, indicating a complex reconfiguration of the oxidative status of the plants rather than a unidirectional mitigation of oxidative damage.

Glycine betaine plays a central role in ROS‐scavenging and membrane‐stabilization (Basit et al. 2025). This occurs alongside the upregulation of CAT in stressed plants (He et al. 2025), which aligns with our observation that pre‐stress treated roots exhibited higher CAT activity and lower MDA levels compared to untreated, drought‐stressed controls (Figure 5B,D). Vaterite‐derived calcium provided additional protection against oxidative injury by maintaining membrane integrity and preventing lipid peroxidation under drought. Adequate Ca^2+^ availability can stabilize membrane phospholipids and proteins against ROS attack, act as a secondary messenger to activate antioxidant enzymes and enhance defence responses, as evidenced by foliar Ca applications boosting antioxidants in other crops (Balantič et al. 2021). This aligns with our observation of sustained or elevated root CAT activity in biostimulant‐treated plants (Figure 5D). *P. protegens* further contributed through induced systemic tolerance, enhancing antioxidant capacity. Wheat inoculated with ACC deaminase‐producing *Pseudomonas* strains showed increased CAT, POD, and SOD activities alongside reduced MDA under stress (Batool et al. 2020; Shahid et al. 2023). Here, biostimulant‐treated plants exhibited moderated MDA accumulation (Figure 5A,B), likely due to *P. protegens*‐induced priming of antioxidative enzymes via hormonal modulation and improved water status (higher relative water content, transpiration), which can indirectly reduce oxidative stress by preventing ROS formation.

The ionomic analysis showed that both pre‐ and post‐stress treatments produced a shoot nutrient profile more similar to that of well‐watered controls than to that of untreated drought‐stressed plants (Figure 6). This pattern indicates that biostimulant application partially attenuated drought‐associated changes in shoot mineral concentrations, a critical factor for preserving overall plant productivity (Baxter et al. 2008) and physiological function under drought (Quagliata et al. 2025). *Pseudomonas* spp., including *P. protegens*, enhance nutrient availability through siderophore‐mediated Fe chelation, P solubilization, and trace element mobilization (Sah et al. 2021). In cereals, *P. fluorescens* inoculation under drought boosts foliar N, P, K, and Ca while reducing oxidative damage (Hyder et al. 2023; Kapoor et al. 2024). In our study, the biostimulant‐treated plants maintained higher levels of Ca, Mg, K, and micronutrients in shoots compared to untreated droughted plants, likely via improved root uptake, reduced stress‐induced senescence, and enhanced transpiration from superior rooting/osmoprotection (Figure 6A). Glycine betaine and calcium also showed complementary roles in this context. Glycine betaine sustained photosynthetic activity and stabilized membrane transporters, maintaining energy‐dependent ion uptake. Calcium, on the other hand, regulated root ion channels, prevented membrane leakiness, and upregulated high‐affinity transport systems via signaling (Patra et al. 2021). Vaterite likely provided sustained foliar Ca^2+^ release, supporting these mechanisms and ensuring efficient nutrient acquisition/retention under stress.

The root ionomic response followed a different pattern, with pre‐D and post‐D plants forming a separate cluster rather than returning to the control profile (Figure 6B). Thus, the treatment did not uniformly restore whole‐plant ionomic homeostasis but generated tissue‐specific changes in nutrient composition.

The contrasting proline responses following pre‐ and post‐stress application further demonstrate that the effects of the biostimulant depended on both treatment timing and tissue. Pre‐stress application increased shoot proline concentration by 77% relative to untreated drought‐stressed plants, whereas post‐stress application decreased root proline concentration by 20% (Figure 3A,B). Proline accumulation has been associated with osmotic adjustment, but it has also been reported to act as a radical scavenger to stabilize proteins, DNA and membranes in plants exposed to stress (Kishor and Sreenivasulu 2013; Signorelli et al. 2013). However, high proline concentrations may also reflect greater stress exposure, and the relationship between proline accumulation and stress tolerance is not always direct. The 77% increase in shoot proline with pre‐stress biostimulant treatment, compared to drought stress alone (Figure 3A), suggested a powerful osmoprotective strategy in the above‐ground tissues. This is further supported by the general increase in *TdP5CS* gene expression in shoots under drought with biostimulant application, indicating an enhanced capacity for proline biosynthesis via the glutamate pathway, a well‐established mechanism for osmotic adjustment and cellular protection during stress (Verbruggen and Hermans 2008; Szabados and Savouré 2010) (Figure 3C). On the other hand, the observed 20% decrease in root proline following post‐stress biostimulant application, alongside a stable root *TdP5CS* expression, could indicate a successful mitigation of stress perception, but it may also reflect changes in proline synthesis, degradation, transport, compartmentation, osmotic demand, or the activation of other protective responses (Figure 3B,D). However, it is crucial to consider that although *TdP5CS* expression and proline levels are often correlated (Luan et al. 2024), discrepancies may point to post‐transcriptional or post‐translational regulation influencing proline availability. For instance, Forlani et al. (2015) observed only minor changes in *P5CS* gene mRNA levels in PEG‐stressed rice ( *Oryza sativa* L.) cells, despite a rapid and significant increase in free proline. In this case, the absence of a corresponding change in root *TdP5CS* expression further indicates that proline concentration cannot be explained solely by transcriptional regulation of this biosynthetic gene.

At the transcriptional level, biostimulant application was associated with lower expression of selected stress‐responsive genes, including *TdDHN15.3* (a dehydrin gene) and *TdDRF1*, in roots and shoots, respectively. These results are consistent with a reduced activation of some drought‐responsive pathways relative to untreated drought‐stressed plants, reasonably suggesting a reduced energetic expenditure on defence or a reallocation of resources toward growth and repair. Such a recalibration of gene expression, prioritizing growth and recovery over defensive responses, aligns with the broader understanding of stress mitigation in plants, as reviewed by Huot et al. (2014). Recently, Song et al. (2024) demonstrated in barley that growth and development pathways are upregulated during recovery from drought, while stress‐related genes are downregulated. This phenomenon is supported by studies showing that biostimulants, including those based on beneficial microorganisms like plant growth‐promoting rhizobacteria, modulate hormonal balance and gene expression, thereby reducing the need for strong stress responses and promoting growth (Barnawal et al. 2017). Additionally, reduced *TdSHN1* expression post‐drought correlates with studies showing biostimulant‐mediated preservation of shoot water content (Turan et al. 2023), potentially reducing cuticular wax dependency. *SHN1* genes are involved in regulating cuticle development and wax accumulation, and are consequently crucial for plant drought tolerance by controlling water loss (Khoudi 2022). Thus, *TdSHN1* suppression suggests that biostimulant may alter cuticular wax biosynthesis pathways during stress adaptation.

The transcriptional response was not uniformly reduced. *TdWRKY2* expression increased in shoots of biostimulant‐treated plants (Figure 4E), despite the general downregulation of other stress‐related genes. This contrasting pattern indicates selective and gene‐specific transcriptional modulation rather than generalized suppression of the drought response. *WRKY* TFs often act as both positive and negative regulators depending on the specific stress and plant species (Li et al. 2024; Wang et al. 2025). While *WRKYs* often regulate stress responses, their upregulation may indicate involvement in specific recovery processes or a unique biostimulant‐signaling pathways, as seen in maize seedlings where drought altered expression profiles of TFs from 37 families (Zhang et al. 2018). However, further research is necessary to fully elucidate the specific function of *TdWRKY2* in this biostimulant‐mediated stress response and the functions of these expression changes during recovery.

Taken together, the gene expression data demonstrate that biostimulant application was associated with tissue‐ and timing‐dependent changes in selected drought‐responsive transcripts. These transcriptional changes coincided with the observed growth and morphological responses, but the present data do not establish causal relationships between gene expression and growth recovery.

## 5 Conclusion

Our findings show that the tested biostimulant was associated with changes in plant growth, root architecture, oxidative markers, nutrient concentrations, and the expression of selected drought‐responsive genes. These effects were strongly dependent on application timing and differed between shoots and roots.

The post‐stress application produced the clearest growth response, restoring shoot biomass to values statistically comparable with those of well‐watered controls. It also increased leaf greenness and stomatal density relative to untreated drought‐stressed plants and shifted the overall root morphological profile toward that of well‐watered controls. In addition, the shoot ionomic profile became more similar to the control profile, although root nutrient composition, oxidative markers, antioxidant activities, and protein concentrations were not uniformly restored.

The response should therefore be interpreted as a timing‐dependent association between biostimulant application and multiple physiological, morphological, ionomic, and transcriptional traits rather than as complete restoration of physiological homeostasis or evidence of a specific mechanism. Under the controlled conditions used here, post‐stress application represents a promising approach for supporting the recovery of drought‐sensitive durum wheat seedlings. However, its agronomic effectiveness remains to be validated under field conditions.

Further studies should investigate the specific bioactive compounds within the biostimulant and integrate hormone profiling, photosynthetic measurements, ROS dynamics, and metabolomics to establish the mechanisms responsible for the observed timing‐dependent responses and to determine their relevance under agronomic conditions.

## Author Contributions

Stefania Astolfi and Gianpiero Vigani contributed to the study conception, design, review, and editing of the manuscript. Material preparation, data collection, and analysis were performed by Matteo Spada, Giulia Quagliata, and Moez Maghrebi. The first draft of the manuscript was written by Matteo Spada and Stefania Astolfi. Eleonora Coppa, Roberto Ruggeri, and Francesco Rossini contributed to the review and editing of the manuscript. All authors have read and approved the final manuscript.

## Funding

This work was supported by Partnership for Research and Innovation in the Mediterranean Area (J83C24000800006).

## Conflicts of Interest

The authors declare no conflicts of interest.

## Supporting information


**Figure S1:** Effect of drought stress and biostimulant application on root morphological traits of durum wheat seedlings grown for 15 days under different growth conditions: C, control plants (adequately supplied with water); T, control plants treated with biostimulant; D, plants subjected to drought; pre‐D and post‐D, plants treated with biostimulant before and after drought imposition, respectively. Each reported value represents the mean ± standard deviation (SD) of measurements performed in triplicate and obtained from three independent biological replicates (*n* = 3). Means sharing the same letter are not significantly different according to Tukey's HSD test (*p* < 0.05).
**Figure S2:** Concentrations of Na (A), Mg (B), K (C), Ca (D), Mn (E), Fe (F), Cu (G), Zn (H), and Mo (I) in shoot and root tissues of durum wheat seedlings grown for 15 days under different growth conditions: C, control plants (adequately supplied with water); T, control plants treated with biostimulant; D, plants subjected to drought; pre‐D and post‐D, plants treated with biostimulant before and after drought imposition, respectively. Each reported value represents the mean ± standard deviation (SD) of measurements performed in triplicate and obtained from three independent biological replicates (*n* = 3). Means sharing the same letter are not significantly different according to Tukey's HSD test (*p* < 0.05).

## Data Availability

The data that support the findings of this study are available on request from the corresponding author. The data are not publicly available due to privacy or ethical restrictions.
