# Supplementary material for: Timing Matters: Post‐Stress Biostimulant Application Promotes Growth Recovery and Physiological Rebalancing in Drought‐Stressed Durum Wheat ( Triticum durum Desf.)
Source: Physiol Plant. 2026 Sep 14;178(5):e71104. doi: 10.1111/ppl.71104 (PMC13575595; doi:10.1111/ppl.71104)
Supplement: Supplementary file 1 [file PPL-178-e71104-s001.docx]

**Timing Matters: post-stress biostimulant application promotes growth recovery and physiological rebalancing in drought-stressed durum wheat (*Triticum durum* Desf.)**

Matteo Spada^1^, Moez Maghrebi^2^, Giulia Quagliata^1^, Eleonora Coppa^1^, Roberto Ruggeri^1^, Francesco Rossini^1^, Gianpiero Vigani^2^, Stefania Astolfi^1*^

^1^Department of Agriculture and Forest Sciences (DAFNE), University of Tuscia, Viterbo, Italy

^2^Department of Life Sciences and Systems Biology, University of Torino, Italy

*Corresponding author: Stefania Astolfi

Suppl. Fig. 1.  Effect of drought stress and biostimulant application on root morphological traits of durum wheat seedlings grown for 15 days under different growth conditions: C, control plants (adequately supplied with water); T, control plants treated with biostimulant; D, plants subjected to drought; pre-D and post-D, plants treated with biostimulant before and after drought imposition, respectively. Each reported value represents the mean ± standard deviation (SD) of measurements performed in triplicate and obtained from three independent biological replicates (n=3). Means sharing the same letter are not significantly different according to Tukey’s HSD test (p < 0.05).

Suppl. Fig. 2. Concentrations of Na (A), Mg (B), K (C), Ca (D), Mn (E), Fe (F), Cu (G), Zn (H), and Mo (I) in shoot and root tissues of durum wheat seedlings grown for 15 days under different growth conditions: C, control plants (adequately supplied with water); T, control plants treated with biostimulant; D, plants subjected to drought; pre-D and post-D, plants treated with biostimulant before and after drought imposition, respectively. Each reported value represents the mean ± standard deviation (SD) of measurements performed in triplicate and obtained from three independent biological replicates (n=3). Means sharing the same letter are not significantly different according to Tukey’s HSD test (p < 0.05).
